# Radiomics based on MRI in predicting lymphovascular space invasion of cervical cancer: a meta-analysis

**DOI:** 10.3389/fonc.2024.1425078

**Published:** 2024-10-17

**Authors:** Chongshuang Yang, Min Wu, Jiancheng Zhang, Hongwei Qian, Xiangyang Fu, Jing Yang, Yingbin Luo, Zhihong Qin, Tianliang Shi

**Affiliations:** ^1^ Department of Radiology, Tongren People’s Hospital, Tongren, China; ^2^ Department of Radiology, Wanshan District People’s Hospital, Tongren, China

**Keywords:** cervical cancer, radiomics, magnetic resonance imaging, systematic review, lymphovascular space invasion

## Abstract

**Objective:**

The objective of this meta-analysis is to assess the efficacy of radiomics techniques utilizing magnetic resonance imaging (MRI) for predicting lymphovascular space invasion (LVSI) in patients with cervical cancer (CC).

**Methods:**

A comprehensive literature search was conducted in databases including PubMed, Embase, Cochrane Library, Medline, Scopus, CNKI, and Wanfang, with studies published up to 08/04/2024, being considered for inclusion. The meta-analysis was performed using Stata 15 and Review Manager 5.4. The quality of the included studies was evaluated using the Quality Assessment of Diagnostic Accuracy Studies 2 and Radiomics Quality Score tools. The analysis encompassed the pooled sensitivity, specificity, positive likelihood ratio (PLR), negative likelihood ratio (NLR), and diagnostic odds ratio (DOR). Summary ROC curves were constructed, and the AUC was calculated. Heterogeneity was investigated using meta-regression. Statistical significance was set at *p* ≤ 0.05.

**Results:**

There were 13 studies involving a total of 2,245 patients that were included in the meta-analysis. The overall sensitivity and specificity of the MRI-based model in the Training set were 83% (95% CI: 77%–87%) and 72% (95% CI: 74%–88%), respectively. The AUC, DOR, PLR, and NLR of the MRI-based model in the Training set were 0.89 (95% CI: 0.86–0.91), 22 (95% CI: 12–40), 4.6 (95% CI: 3.1–7.0), and 0.21 (95% CI: 0.16–0.29), respectively. Subgroup analysis revealed that the AUC of the model combining radiomics with clinical factors [0.90 (95% CI: 0.87–0.93)] was superior to models based on T2-weighted imaging (T2WI) sequence [0.78 (95% CI: 0.74–0.81)], contrast-enhanced T1-weighted imaging (T1WI-CE) sequence [0.85 (95% CI: 0.82–0.88)], and multiple sequences [0.86 (95% CI: 0.82–0.89)] in the Training set. The pooled sensitivity and specificity of the model integrating radiomics with clinical factors [83% (95% CI: 73%–89%) and 86% (95% CI: 73%–93%)] surpassed those of models based on the T2WI sequence [79% (95% CI: 71%–85%) and 72% (95% CI: 67%–76%)], T1WI-CE sequence [78% (95% CI: 67%–86%) and 78% (95% CI: 68%–86%)], and multiple sequences [78% (95% CI: 67%–87%) and 79% (95% CI: 70%–87%)], respectively. Funnel plot analysis indicated an absence of publication bias (*p* > 0.05).

**Conclusion:**

MRI-based radiomics demonstrates excellent diagnostic performance in predicting LVSI in CC patients. The diagnostic performance of models combing radiomics and clinical factors is superior to that of models utilizing radiomics alone.

**Systematic review registration:**

https://www.crd.york.ac.uk/PROSPERO/#myprospero, identifier CRD42024538007.

## 1 Introduction

Cervical cancer (CC) ranks as the fourth most prevalent cancer among women globally. In 2020, statistics reported approximately 604,000 new cases and 342,000 deaths worldwide (1, 2). Lymphovascular space invasion (LVSI), as the name indicates, occurs when cancer cells invade the blood vessels or lymphatics. This process is a critical step in the metastasis of cancer cells to other locations or organs (3). The National Comprehensive Cancer Network (NCCN) guidelines for CC consider LVSI a critical determinant in selecting appropriate treatment plans. Early-stage CC with or without LVSI requires markedly different therapeutic approaches. For CC patients with International Federation of Gynecology and Obstetrics (FIGO) IA1 stage, conization is recommended for tumors without LVSI. However, patients with LVSI, even at the IA1 stage, require radical hysterectomy and pelvic lymph node dissection. Therefore, preoperative determination of LVSI plays a pivotal role in patient surgical planning and systemic treatment (4).

Magnetic resonance imaging (MRI) is the most commonly used imaging methods for CC evaluation, and MRI-based assessment has significantly improved the accuracy of CC diagnosis in recent decades (5). Although functional MRI techniques, including dynamic contrast-enhanced MRI (DCE-MRI), amide proton transfer imaging, and diffusion-weighted imaging (DWI), have been employed for predicting LVSI in CC (6, 7), the accuracy of conventional MRI based on subjective visual assessment by radiologists remains low.

Radiomics, an approach that extracts quantitative features from imaging regions of interest in an automated and high-throughput manner, can quantify tumor heterogeneity, including tumor cell density, extracellular matrix deposition, angiogenesis, and necrosis degree, thereby reflecting tissue characteristics (8). In recent years, MRI-based radiomics has been widely applied to predict pathological type, grade, parametrial invasion, LVSI, and lymph node metastasis in CC (9–12). These outstanding results suggest that MRI-based radiomics may serve as an accurate and non-invasive tool for evaluating CC by analyzing primary tumors preoperatively.

Current studies on MRI-based radiomics for assessing LVSI in CC are predominantly single-center investigations with limited sample sizes, resulting in substantial heterogeneity in the reported diagnostic performance parameters. Studies conducted by Wang et al. (13) showed that the sensitivity of MRI-based radiomics in predicting LVSI in CC was as high as 94.2%, while Cui et al. (14) reported a sensitivity of only 58.1%. Furthermore, the overall diagnostic performance of MRI-based radiomics in predicting LVSI in CC has not yet been systematically evaluated. Therefore, the purpose of this meta-analysis was to determine the diagnostic performance of radiomics based on preoperative MRI in predicting LVSI in CC.

## 2 Methods

This study was conducted in accordance with the Preferred Reporting Items for Systematic Reviews and Meta-Analyses (PRISMA) guidelines (15). The evidence-based analysis was prospectively registered in PROSPERO. Two researchers (Yang CS and Wu M) independently performed each step of the analysis, engaging in discussion to reach a consensus in case of disagreements.

### 2.1 Search strategy

Systematic searches were conducted in Embase, PubMed, Cochrane Library, Medline, Scopus, Wanfang, and CNKI databases up to 08/04/2024. The search formula was as follows: [(“Uterine Cervical Neoplasms”[Mesh]) OR (Cervical Neoplasm, Uterine) OR (Neoplasm, Uterine Cervica) OR (Uterine Cervical Neoplasm) OR (Neoplasms, Cervical) OR (Cervical Neoplasms) OR (Cervical Neoplasm) OR (Neoplasms, Cervix) OR (Cervix Neoplasm) OR (Neoplasm, Cervix) OR (Cervix Neoplasms) OR (Cancer of the Uterine Cervix) OR (Cancer of the Cervix) OR (Cervical Cancer) OR (Cancer, Cervical) OR (Cervical Cancers) OR (Uterine Cervical Cancer) OR (Cancer, Uterine Cervical) OR (Cervical Cancer, Uterine) OR (Uterine Cervical Cancers) OR (Cancer of Cervix) OR (Cervix Cancer) OR (Cancer, Cervix)] AND [(magnetic resonance imaging) OR (MRI) OR (MR)] AND [(radiomic) OR (machine learning) OR (deep learning) OR (artificial intelligence) OR (texture)]. Additionally, the reference lists of the included studies were examined to identify further eligible publications.

### 2.2 Inclusion and exclusion criteria

The inclusion criteria encompassed peer-reviewed publications in Chinese and English that met the following conditions: utilization of biopsy or surgical pathology results as the gold standard; employing radiomics based on MRI as the index test for assessing LVSI in CC; ensuring blinding of radiologists and pathologists; and possessing the ability to calculate true positives, false positives, true negatives, and false negatives in both Training and Validation sets. Exclusion criteria comprised animal or laboratory studies, case reports, conference reports, comments, and responses.

### 2.3 Literature screening and data extraction

Duplicate publications were excluded using EndNote 21 software. The titles and abstracts of the remaining articles were thoroughly scrutinized to eliminate reviews, conference abstracts, and individual case reports. A comprehensive examination of the screened articles was performed to exclude studies that did not focus on MRI-based radiomics in predicting LVSI of CC or lacked extractable data. Furthermore, when multiple articles utilized data from the same set, only the study containing the largest number of cases and the most detailed information was included in this meta-analysis. This meticulous process resulted in the final selection of included publications.

Relevant information was meticulously extracted from each selected article, including the first author, publication year, type of research, MRI machine manufacturer and magnetic field strength, MRI sequences, segmentation details, total patient population (encompassing both training and Validation sets), method of radiomic feature selection, FIGO stage, true positive, true negative, false positive, and false negative values.

If an article reported multiple models, the model with the largest sum of sensitivity and specificity in the Training set was considered the best model for that article. Subsequently, subgroups (T2WI sequence model, T1WI-CE sequence model, multiple-sequence model, and radiomics combined with clinical factor model) were defined based on the MRI sequences from which radiomic features were extracted.

### 2.4 Quality assessment

Two reviewers independently assessed the methodological quality and risk of bias of the included studies using the Quality Assessment of Diagnostic Accuracy Studies 2 (QUADAS-2) (16) and Radiomics Quality Score (RQS) (8) guidelines, respectively.

### 2.5 Meta-analysis

Meta-analysis was performed using Stata 15 (Stata Corp, College Station, TX) and Review Manager 5.4. Pooled sensitivity, specificity, DOR, positive and negative likelihood ratios (PLR and NLR), and AUC, with corresponding 95% confidence intervals (CIs), were calculated. A forest plot and a summary receiver operating characteristic (SROC) plot were generated. Heterogeneity among study results was analyzed using the chi-square test, and the extent of heterogeneity was quantitatively determined. Deek’s funnel plot was employed to detect the presence of publication bias. *p* < 0.05 was considered statistically significant.

## 3 Results

### 3.1 Literature screening

**Figure 1:**
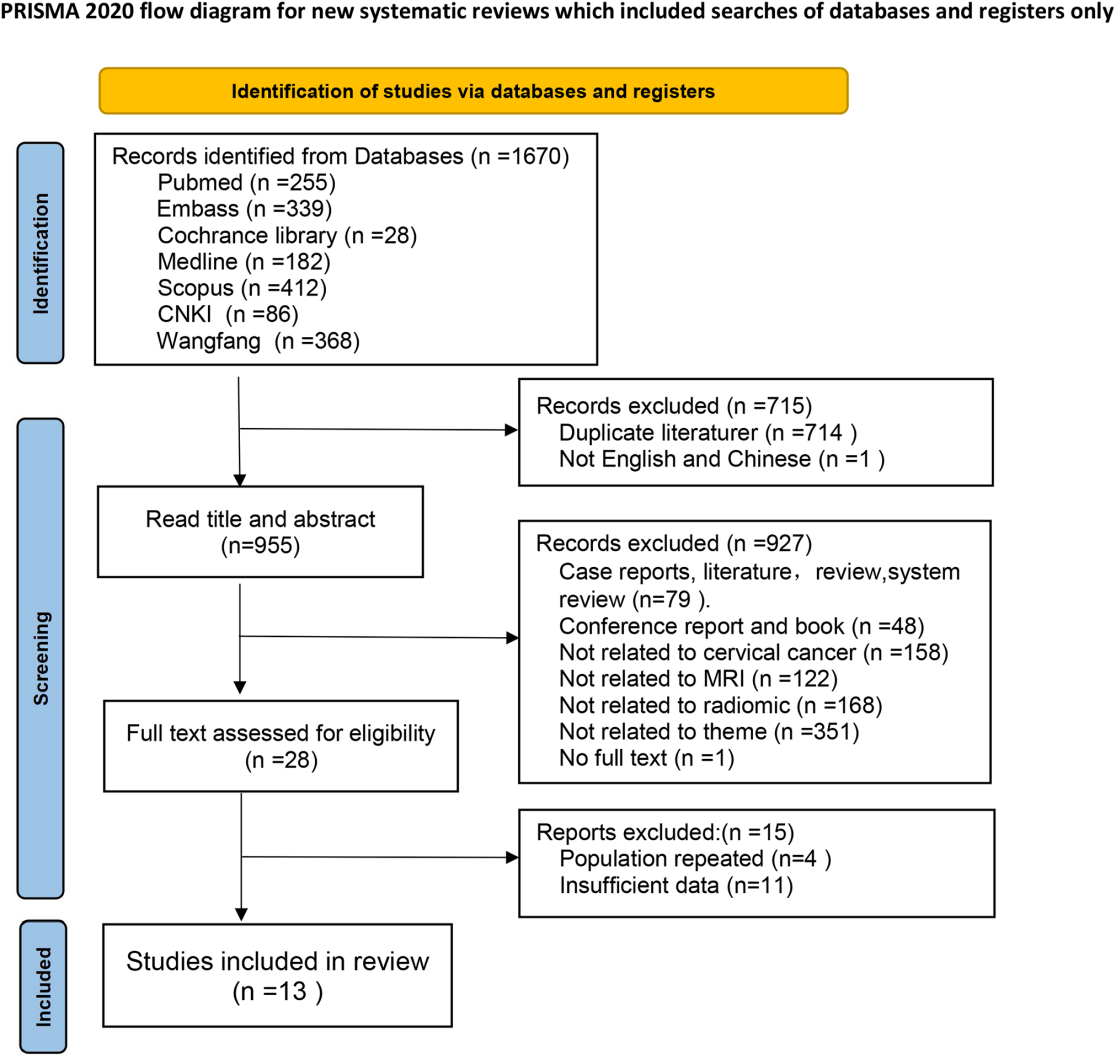
Flow diagram illustrating the process for selecting studies.

The electronic search yielded a total of 1,670 potentially eligible citations, of which 714 studies were duplicates and 1 study was not in Chinese or English language. Subsequently, 927 studies were excluded after the assessment of title and abstract. Following a careful evaluation of the full text, an additional 15 studies were excluded. Finally, 13 articles were included, comprising 4 studies in Chinese and 9 studies in English. The flowchart of the study screening process is depicted in **Figure 1**.

### 3.2 Basic features of studies

All studies were retrospective in nature. ROIs of all studies were 3D and manually segmented. The total sample size of the studies ranged from 110 to 300. Patients in two studies were from multiple centers. MRI sequences used in all studies included T2WI-Axis, T2WI-FS-Axis, T2WI-sag, T1WI-Axis, T1WI-CE-Axis, DWI-Axis, ADC-Axis, and combinations of these sequences. Eight articles (14, 17–23) reported radiomic models based on T2WI sequences (four articles with T2WI-Sag, three articles with T2WI-Axis, and one article with T2WI-FS-Axis). Seven articles (3, 13, 14, 18, 19, 21, 22) reported radiomic models based on T1WI-CE sequences (five articles with T1WI-CE-Axis, two articles with T1WI-CE-Sag). Six articles (18, 19, 21, 22, 24, 25) reported radiomic models based on multiple sequences. Seven studies (3, 14, 21, 23–26) reported radiomic models based on the combination of radiomic features and clinical factors. Deep learning was utilized to diagnose LVSI of CC in two studies. The basic features of the included studies are presented in **Table 1**.

**Table 1:** The basic information of literatures.

Author (years)	Type of research	MRI	Sequence	Sample size	Training set	Validation set	Segmentation	ROI	Radiomics feature reduction	FIGO staging
He YM (2022) (20)	Retro	Philips, 3.0 T	T2WI-sag	110	77	33	Manually	3D	LASSO, LR	IB-IIA
Jia YJ (2023) (26)	Retro	Philips, 3.0 T	T2WI-axis, ADC-axis, T1WI-CE-axis	168	117	51	Manually	3D	mRMR LASSO	IB-IIA
Lin BJ (2024) (23)	Retro	Siemens, 3.0 T	T2WI-sag	178	142	36	Manually	3D	LASSO	NA
Wang HB (2021) (19)	Retro	Siemens, 3.0 T	T2WI-FS-axis, DWI-axis, T1WI-CE-sag	134	91	43	Manually	3D	LASSO	IA-IIA
Yu HQ (2022) (21)	Retro	GE, 3.0 T	T2WI-axis, T1WI-CE-axis	123	87	36	Manually	3D	mRMR LASSO	IA-IIIC
Cui LP (2022) (14)	Retro	Siemens, 3.0 T	T2WI-axis, T1WI-CE-axis	163	108	55	Manually	3D	LASSO, LR	IA-IIA
Hu QM. (2022) (22)	Retro	Siemens, 1.5/3.0 T	T2WI-axis, T1WI-axis, T1WI-CE-axis, DWI-axis	276	195	81	Manually	3D	mRMR, LASSO	IA-IB
Hua QW, 2022 (18)	Retro	Siemens, 3.0 T	T1WI-CE-axis、T2WI-sag	167	111	56	Manually	3D	SVM	NA
Li ZC (2019) (3)	Retro	GE, 1.5 T	T1WI-CE-axis	105	70	35	Manually	3D	Univariate LR, mRMR	NA
Wang S. (2019) (17)	Retro	NA	T2WI-axis	120	80	40	Manually	3D	LASSO	IB-IIB
Wang SX (2023) (13)	Retro	Siemens 1.5/3.0 T	T1WI-CE-axis	300	198	102	Manually	3D	LASSO	I-III
Wu Y. (2023) (25)	Retro	Siemens, 1.5/3.0 T	T1WI-axis, T2WI-FS-axis, T1WI-CE-axis	168	129	39	Manually	3D	Spearman, LASSO, LR	IB-IIB
Xiao ML. (2022) (24)	Retro	Siemens 1.5 T	T1WI-axis, T2WI-FS-axis, T1WI-CE-axis, DWI-axis, ADC-axis	233	154	79	Manually	3D	LASSO	IB-IIB

Retro, retrospective; D, dimensionality; T, Tesla; T1WI, T1-weighted imaging; T2WI, T2-weighted imaging; T2WI-FS, T2-weighted imaging fat suppression; T1WI-CE, contrast-enhanced T1 weighted imaging; SVM, support vector machine; LR, logistic regression; DWI, diffusion-weighted imaging; ADC, apparent diffusion coefficient; LASSO, least absolute shrinkage and selection operator; mRMR, max-relevance and min-redundancy; NA, have no data.

### 3.3 Quality of literature

The total RQS was 36, and the RQS of the articles included in this meta-analysis ranged from 12 to 16, with an average of 13.54 ± 1.33. All studies were retrospective in design, and no studies analyzed cost-effectiveness or biological correlates. The detailed RQS is shown in **Table 2**.

**Table 2:** Radiomics quality scores of the included studies.

Study criteria	He YM (2022) (20)	Jia YJ (2023) (26)	Lin BJ (2024) (23)	Wang HB (2021) (19)	Yu HQ (2022) (21)	Cui LP (2022) (14)	Hu QM. (2022) (22)	Hua WQ (2022) (18)	Li ZC (2022) (3)	Wang S. (2019) (17)	Wang SX. (2023) (13)	Wu Y. (2023) (25)	Xiao ML. (2022) (24)
Image protocol quality	1	1	1	1	1	1	1	1	1	1	1	1	1
Multiple segmentations	0	1	0	1	1	1	1	1	0	0	0	1	1
Phantom study	1	1	1	1	1	1	1	1	1	1	1	1	1
Imaging at multiple time points	0	0	0	0	0	0	0	0	0	0	0	0	0
Feature reduction	3	3	3	3	3	3	3	3	3	3	3	3	3
Multivariable analysis	1	1	1	1	1	1	1	1	1	1	1	1	1
Biological correlates	0	0	0	0	0	0	0	0	0	0	0	0	0
Cutoff analysis	0	0	0	0	0	0	0	0	0	0	0	0	0
Discrimination statistics	0	0	0	0	0	0	0	0	0	0	0	0	0
Calibration statistics	0	0	0	0	0	0	0	0	0	0	0	0	0
Prospective study	0	0	0	0	0	0	0	0	0	0	0	0	0
Validation	1	1	1	1	1	1	1	1	1	1	2	2	1
Gold standard	2	2	2	2	2	2	2	2	2	2	2	2	2
Potential clinical applications	2	2	2	2	2	2	2	2	2	2	2	2	2
Cost-effectiveness analysis	0	0	0	0	0	0	0	0	0	0	0	0	0
Open Science and date	1	1	1	1	1	1	3	3	3	1	1	3	3
Total score	12	13	12	13	13	13	15	15	14	12	13	16	15

**Figure 2:**
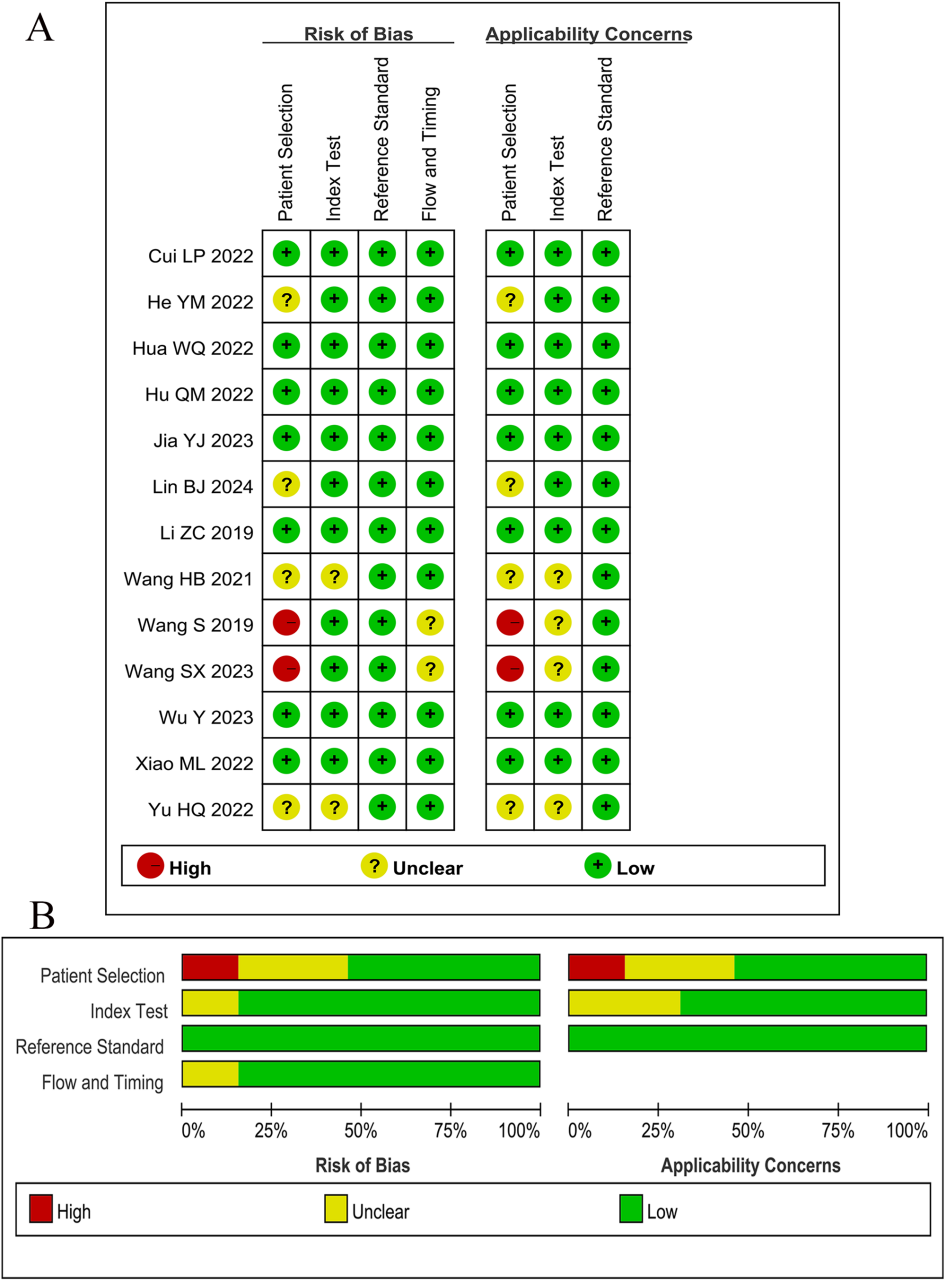
Results of Quality Assessment of Diagnostic Accuracy Studies-2 (QUADAS-2). **(A)** Bias risk assessment results of included studies. **(B)** Risk of bias and applicability concerns summary.

According to QUADAS-2 guidelines, two articles scored high risk of bias in the patient selection field due to the absence of reporting on continuous enrollment and random sampling. Four articles scored unclear risk of bias in the patient selection field as inappropriate exclusions were not reported. All other indicators exhibited low risk. Overall, the methodological quality of the included studies was at a medium level. The methodological quality of the studies is illustrated in **Figure 2**.

### 3.4 Diagnostic value of the best radiomic model

**Figure 3:**
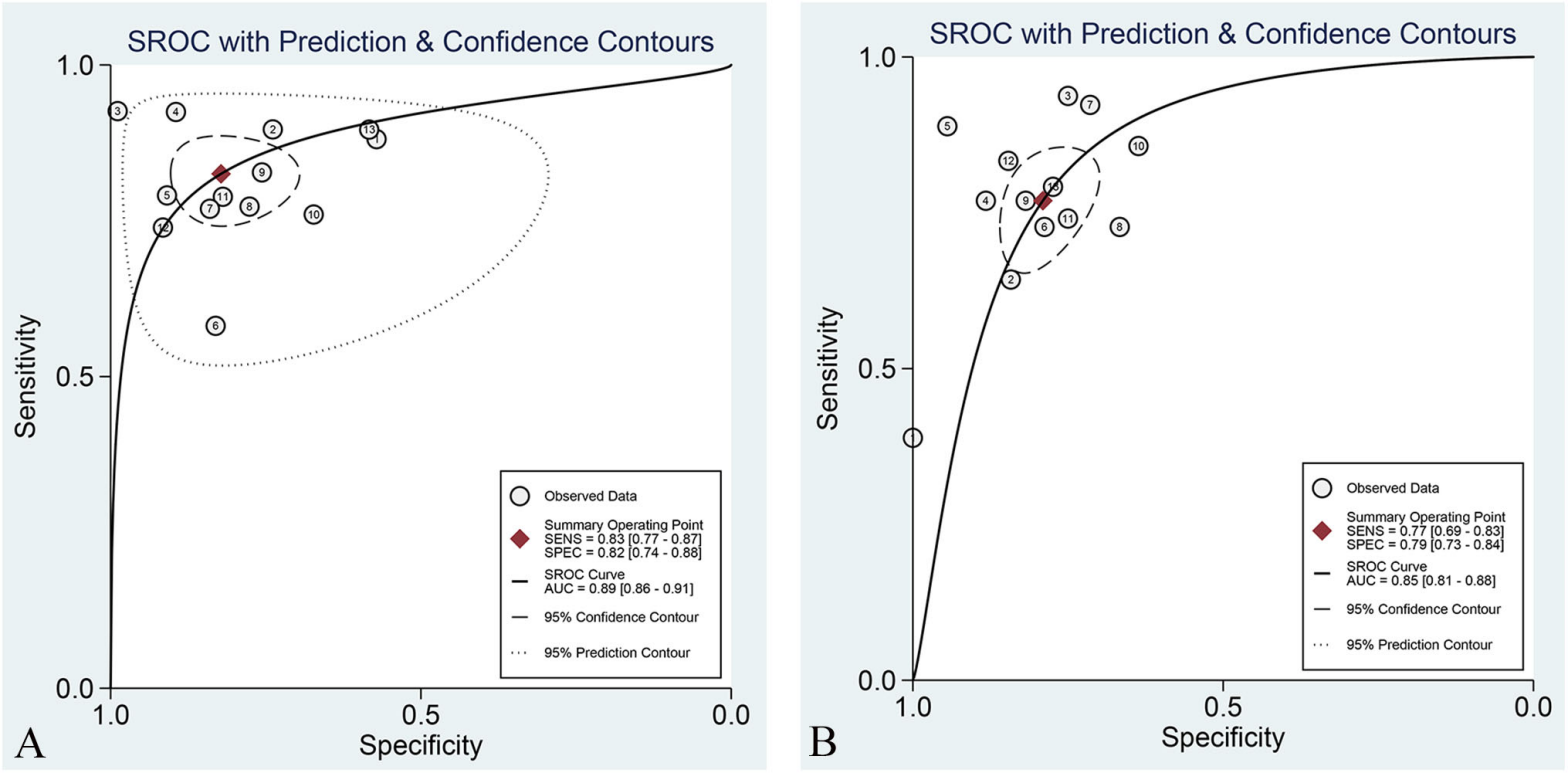
Forest plot of sensitivity and specificity of the best radiomic model based on MRI for predicting LVSI of CC in the Training set **(A)** and Validation set **(B)**, respectively.

**Figure 4:**
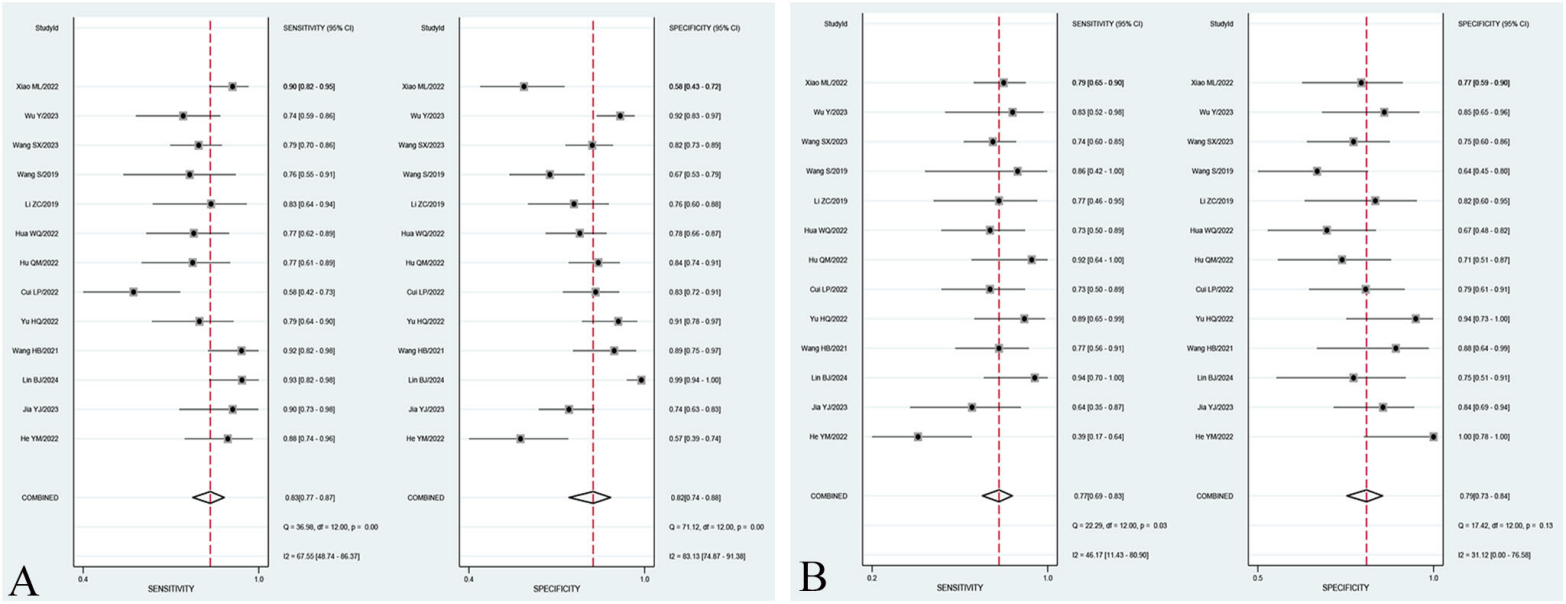
SROC curve of the best radiomic model based on MRI for predicting LVSI of CC in the Training set **(A)** and Validation set **(B)**.

The overall sensitivity and specificity of the Training set and Validation set were 83% (95% CI: 77%–87%), 72% (95% CI: 74%–88%), and 77% (95% CI: 69%–83%), 79% (95% CI: 73%–84%), respectively. AUC, DOR, PLR, and NLR of the Training set and Validation set were 0.89 (95% CI: 0.86–0.91), 22 (95% CI: 12–40), 4.6 (95% CI: 3.1, 7.0), 0.21 (95% CI: 0.16–0.29), and 0.85 (95% CI: 0.81–0.85), 13 (95% CI: 8.0–19), 3.7 (95% CI: 2.9–4.7), and 0.29 (95% CI: 0.22–0.39), respectively. The forest plots of sensitivity and specificity are presented in **Figure 3**, and the SROC curve is depicted in **Figure 4**.

### 3.5 Subgroup analysis

**Figure 5:**
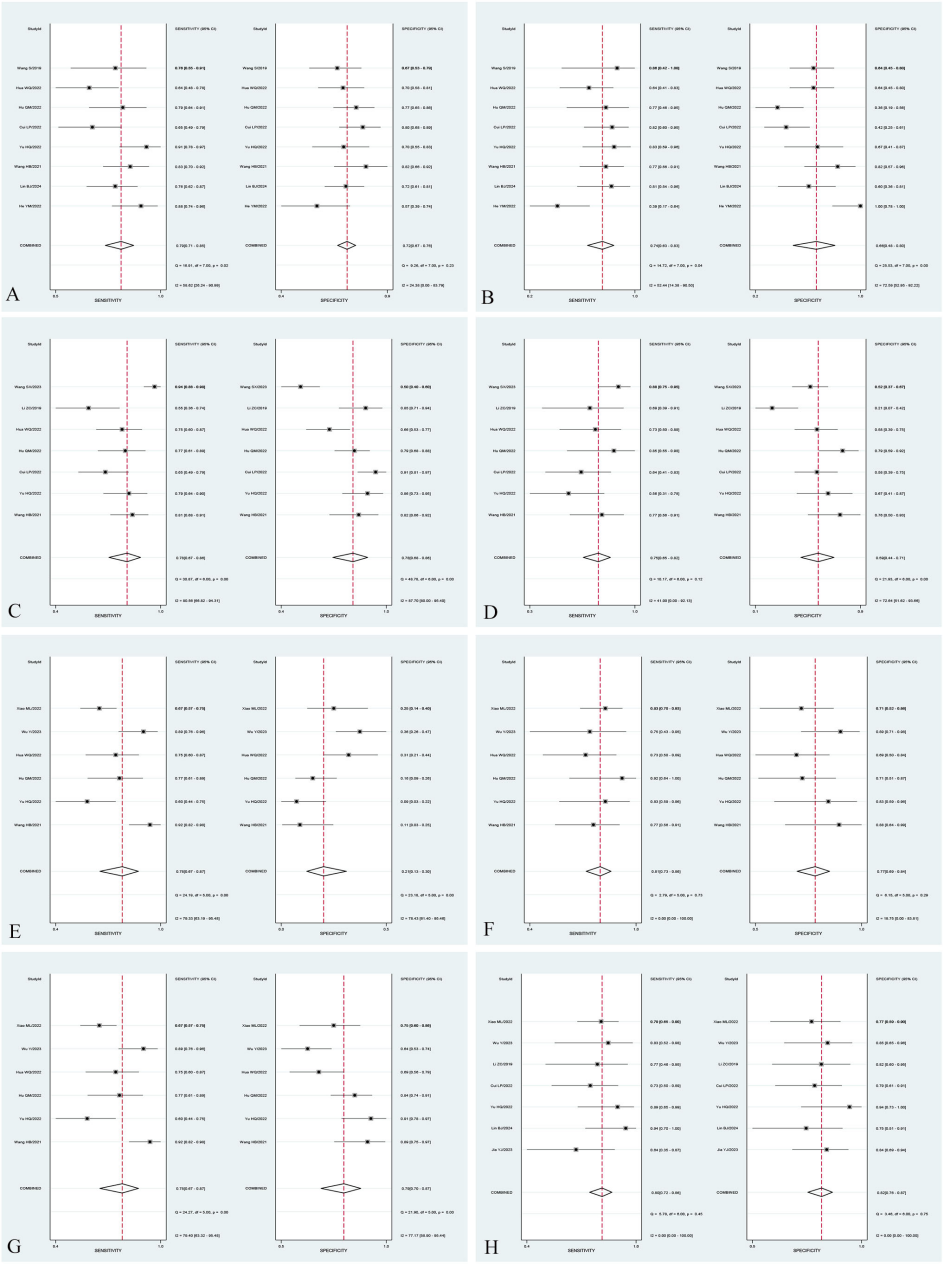
Forest plot of sensitivity and specificity of different subgroups. Forest plot of sensitivity and specificity of radiomic models based on T2WI sequences in the Training set **(A)** and Validation set **(B)**. Forest plot of sensitivity and specificity of radiomic models based on T1WI-CE sequences in the Training set **(C)** and Validation set **(D)**. Forest plot of sensitivity and specificity of radiomic models based on multiple sequences in the Training set **(E)** and Validation set **(F)**. Forest plot of sensitivity and specificity of models based on radiomics combined with clinical factors in the Training set **(G)** and Validation set **(H)**.

In the subgroup analysis, the area under the curve (AUC) of radiomics combined with clinical models [0.90 (95% CI: 0.87–0.93)] was higher than that of T2WI sequence models [0.78 (95% CI: 0.74–0.81)], T1WI-CE sequence models [0.85 (95% CI: 0.82–0.88)], and multiple sequence models [0.86 (95% CI: 0.82–0.89)] in the Training set. The pooled sensitivity of radiomics combined with clinical models [83% (95% CI: 73%–89%)] was superior to that of T2WI sequence models [79% (95% CI: 71%–85%)], T1WI-CE sequence models [78% (95% CI: 67%–86%)], and multiple-sequence models [78% (95% CI: 67%–87%)] in the Training set. Furthermore, the pooled specificity of radiomics combined with clinical models [86% (95% CI: 73%–93%)] outperformed that of T2WI sequence models [72% (95% CI: 67%–76%)], T1WI-CE sequence models [78% (95% CI: 68%–86%)], and multiple-sequence models [79% (95% CI: 70%–87%)] in the Training set. The comprehensive results of the subgroup analysis are presented in **Table 3**, and the corresponding subgroup analysis plots are illustrated in **Figure 5**.

**Table 3:** The results of subgroup analysis.

Model	No.	AUC (95% CI)	Sensitivity (95% CI)	Specificity (95% CI)	PLR	NLR	DOR
Training set							
T2WI	8	0.78(0.74-0.81)	0.79(0.71-0.85)	0.72(0.67-0.76)	2.8	0.30	10
T1WI-CE	7	0.85(0.82-0.88)	0.78(0.67-0.86)	0.78(0.68-0.86)	3.6	0.28	13
Multiple sequence	6	0.86(0.82-0.89)	0.78(0.67-0.87)	0.79(0.70-0.87)	3.8	0.27	14
Radiomics+clinical	7	0.90(0.87-0.93)	0.83(0.73-0.89)	0.86(0.73-0.93)	5.9	0.20	29
Validation set							
T2WI	8	0.77(0.73-0.80)	0.74(0.63-0.83)	0.66(0.48-0.80)	2.2	0.39	6
T1WI-CE	7	0.74 (0.70-0.78	0.75(0.65-0.82)	0.59(0.44-0.71)	1.8	0.43	4
Multiple sequence	6	0.84(0.81-0.87)	0.81(0.73-0.86)	0.77(0.69-0.84)	3.6	0.25	14
Radiomics+clinical	7	0.88(0.85-0.90)	0.80(0.72-0.86)	0.82(0.76-0.87)	4.4	0.25	18

### 3.6 Heterogeneity evaluation

The I^2^ statistic indicated that the overall heterogeneity for sensitivity and specificity in the Training set and Validation set were 67.55%, 81.33% and 46.17%, 31.12%, respectively. The heterogeneity for sensitivity and specificity of studies (Training set) with T2WI sequence models, T1WI-CE sequence models, multiple-sequence models, and radiomics combined with clinical models were 29% and 0%, 80.56% and 87.7%, 79.33% and 78.43%, and 79.40% and 77.17%, respectively. In the Validation set, the heterogeneity for sensitivity and specificity of studies with T2WI sequence models, T1WI-CE sequence models, multiple-sequence models, and radiomics combined with clinical models were 52.44% and 72.59%, 41% and 72.64%, 0% and 18.75%, and 0% and 70%, respectively.

### 3.7 Publication bias

**Figure 6:**
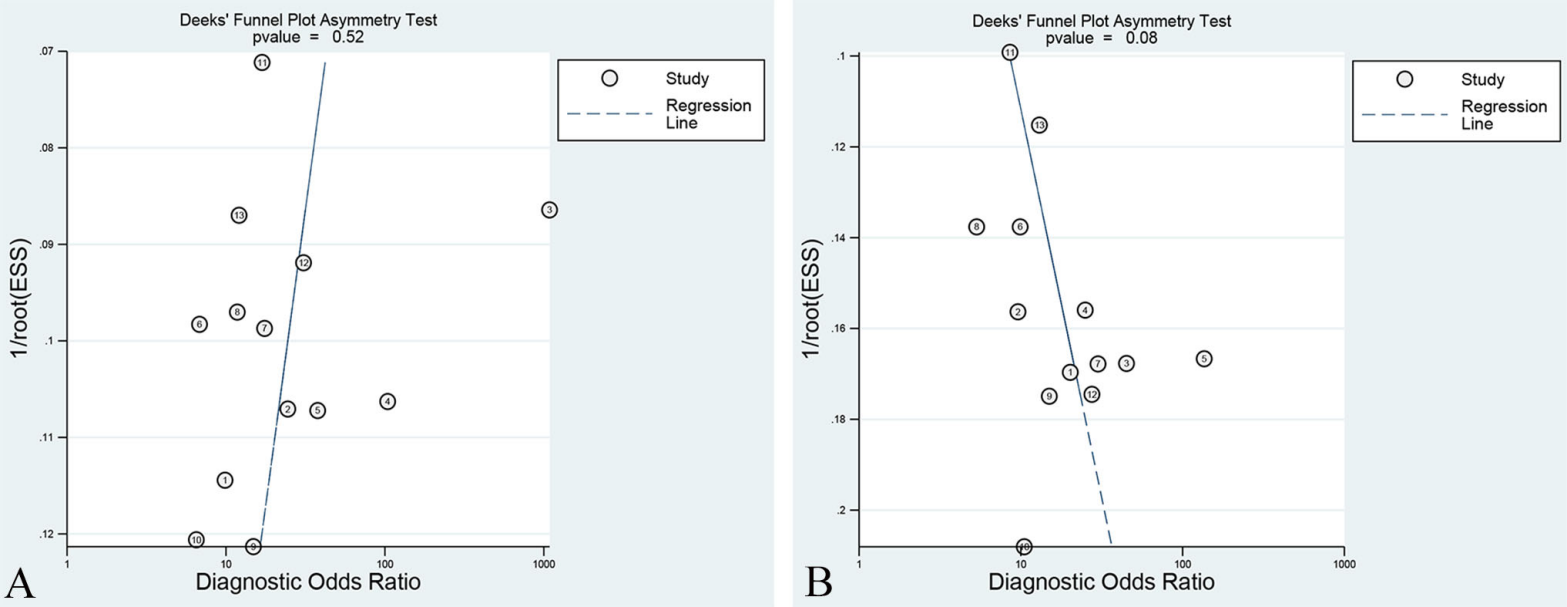
Deek’s funnel plot of the best radiomic model in the Training set **(A)** and Validation set **(B)**.

Deek’s funnel plots were constructed for the included studies to assess potential publication bias. The results demonstrated that overall studies distinguishing LVSI positive and negative exhibited an approximately symmetrical distribution around the central axis. The *p*-values of the Training set and Validation set were 0.52 and 0.08, respectively (presented in **Figure 6**). In the subgroup analysis, the *p*-values of the Training set and Validation set in studies with T2WI sequence models, T1WI-CE sequence models, multiple-sequence models, and radiomics combined with clinical models were 0.53 and 0.25, 0.64 and 0.53, 0.45 and 0.14, and 0.13 and 0.24, respectively. All *p*-values exceeded the 0.05 threshold, suggesting no significant evidence of publication bias.

## 4 Discussion

LVSI is a microscopic characteristic of tumors and is an important independent predictor of poor prognosis in CC (27). Previous studies have shown that the 5-year survival rate of CC patients with LVSI is significantly lower than that of patients without LVSI (28, 29). Routine MRI, including DWI (30), intravoxel incoherent motion MRI (31), and DCE-MRI (32), has been used to predict LVSI of CC before surgery based on the naked-eye observation and diagnostic experience of radiologists. However, the diagnostic efficacy of LVSI based on routine MRI is low and cannot meet clinical requirements.

With the development of medical imaging technology, radiomics has found wide applications in predicting LVSI of cervical cancer. This study adopts a meta-analysis method to summarize the value of radiomics based on MRI in assessing LVSI of CC. The results of this study show that the comprehensive analysis indices of MRI radiomics evaluation for estimating LVSI of cervical cancer, combining sensitivity and specificity, were 83% (95% CI: 77%–87%) and 72% (95% CI: 74%–88%), respectively. The area under the SROC curve was 0.89 (95% CI: 0.86–0.91), suggesting that radiomics based on MRI is an effective, non-invasive, and reliable method for predicting LVSI of CC. Deek’s funnel plot shows no publication bias, suggesting that the results of this study are reliable. Similar meta-analyses have also demonstrated the capability of radiomics based on MRI in assessing LVSI of endometrial carcinoma (33).

In addition, in the subgroup analyses, we found that the AUC values of studies using radiomics combined with clinical factor models [0.90 (0.87–0.93)] to assess LVSI of CC were higher than those of T2WI sequence models [0.78 (0.74–0.81)], T1WI-CE sequence models [0.85 (0.82–0.88)], and multiple-sequence models [0.86 (0.82–0.89)]. Previous studies indicated that lymph node metastasis and high FIGO stage are independent risk factors for predicting LVSI in CC (34). Therefore, the diagnostic accuracy of radiomic models may increase when clinical features are added in detecting LVSI. A meta-analysis conducted by Li et al. (35) also showed that the diagnostic accuracy of radiomic models in which clinical factors were added increased significantly in detecting lymph node metastasis.

Different MRI sequences reflect different aspects of tumor information. In theory, the diagnostic performance of multisequence models, which collect multiple-diameter information from different sequences and reflect tumor information more comprehensively, should be better than that of single-sequence models (19). However, in our study, the pooled sensitivity and specificity of T2WI sequence models, T1WI-CE sequence models, and multiple-sequence models showed no significant difference. A possible reason is that the heterogeneity for sensitivity and specificity of studies was high, with a wide range (from 0% to 87.7%). This was attributed to the inconsistent stages of the patients included in the studies (some studies included patients with stage IIA and below, some included patients with stage IIB and above, and some studies did not clearly state the stage of the patients). In addition, all of the included studies were retrospectively designed. The potential risk of selective reporting of positive results is unavoidable due to the lack of predetermined study protocols. Therefore, this finding also requires well-designed and appropriate prospective randomized trials to demonstrate its validity (36, 37).

This meta-analysis has several limitations that should be acknowledged. Firstly, the original studies included in this meta-analysis have small sample sizes, which may introduce potential small sample size effects. Moreover, the high heterogeneity of individual indicators may decrease the reliability of the results to some extent. Secondly, our study only includes literature published in Chinese and English, which may introduce language bias. Third, our study did not perform subgroup analysis on studies using different equipment (Siemens, GE, and Philips) or different magnetic field strengths (3.0 T, 1.5 T).Despite these limitations, this study represents the first meta-analysis worldwide to examine the value of MRI-based radiomics in predicting LVSI of CC. The findings of this paper support the conclusions of previous studies, demonstrating that MRI-based radiomics can be effectively used to predict LVSI of CC. Furthermore, this study provides the latest and most comprehensive evidence-based medical evidence for the clinical diagnosis of CC using MRI-based radiomics.

## 5 Conclusion

In conclusion, this meta-analysis demonstrates the value of MRI-based radiomics in the preoperative prediction of LVSI of CC. The diagnostic performance of models combining radiomics and clinical factors was found to be superior to that of radiomics alone. However, due to the high heterogeneity among the included articles, no significant difference in diagnostic performance was observed between models based on single MRI sequences and those based on multisequence data. This finding still needs to be further verified through larger-scale, prospective studies to confirm its validity and generalizability.

## Data Availability

The original contributions presented in the study are included in the article/supplementary material. Further inquiries can be directed to the corresponding author.
